# Relationship between Tibial Baseplate Design and Rotational Alignment Landmarks in Primary Total Knee Arthroplasty

**DOI:** 10.1155/2015/189294

**Published:** 2015-09-28

**Authors:** Pier Francesco Indelli, Angelo Graceffa, Andrea Baldini, Brielle Payne, Gennaro Pipino, Massimiliano Marcucci

**Affiliations:** ^1^Department of Orthopaedics and Rehabilitation, University of New Mexico and New Mexico Veterans Affairs Health Care System (NMVAHCS), Albuquerque, NM 87108, USA; ^2^Clinica Ortopedica, Universita degli Studi di Firenze, CESAT, 50054 Fucecchio, Italy; ^3^IFCA, 50139 Firenze, Italy; ^4^UNILUDES, 6900 Lugano, Switzerland

## Abstract

This study evaluated the influence of modern tibial baseplate designs when using the anterior tibial cortex as a primary rotational landmark for the tibial baseplate in TKA. Eighty patients undergoing TKA were randomized in two groups. Group 1 included 25 females and 15 males receiving a posterior-stabilized (PS) symmetric tibial baseplate while Group 2 included 24 females and 16 males receiving a PS anatomical tibial component. Identical surgical technique, including the use of the surgical transepicondylar femoral axis (sTEA) and the anterior tibial cortex (“Curve-on-Curve”) as rotational alignment landmarks, was used. All patients underwent CT evaluation performed with the knee in full extension. Three observers independently measured the rotational alignment of the tibial component in relation to the sTEA. The rotational alignment of the symmetric baseplate showed an average external rotation of 1.3° (minimum 5°, maximum −1°): 91% of the knees showed 0 ± 3° with respect to the surgical sTEA, being internally rotated in 20%. The rotational alignment of the anatomical baseplate showed an average external rotation of 4.1° (minimum 0.4°, maximum 8.9°): only 47.5% of the knees showed 0 ± 3°, being externally rotated in 100%. The difference between the two groups was statistically significant. This study confirms the reliability of the “Curve-on-Curve” technique as an adequate rotational alignment anatomical landmark in TKA: the use of an asymmetric tibial baseplate might lead to external rotation of the tibial component when this technique is intraoperatively chosen.

## 1. Introduction

Many studies related a total knee arthroplasty (TKA) poor functional outcome to rotational malalignment of the femoral and tibial components [1–3]. The goal of tibial component rotational alignment in primary TKA is to achieve, on the coronal plane, parallelism between the femoral transepicondylar axis (TEA) and the mediolateral (ML) axis of the tibial component, avoiding errors in internal or external rotation between the two axes. This desired coronal parallelism during active range-of-motion (AROM) is hard to be achieved because the TEA has been demonstrated to be cylindrical [4] and the tibial plateau undergoes a substantial internal rotation during ROM [5].

**Figure 1:**
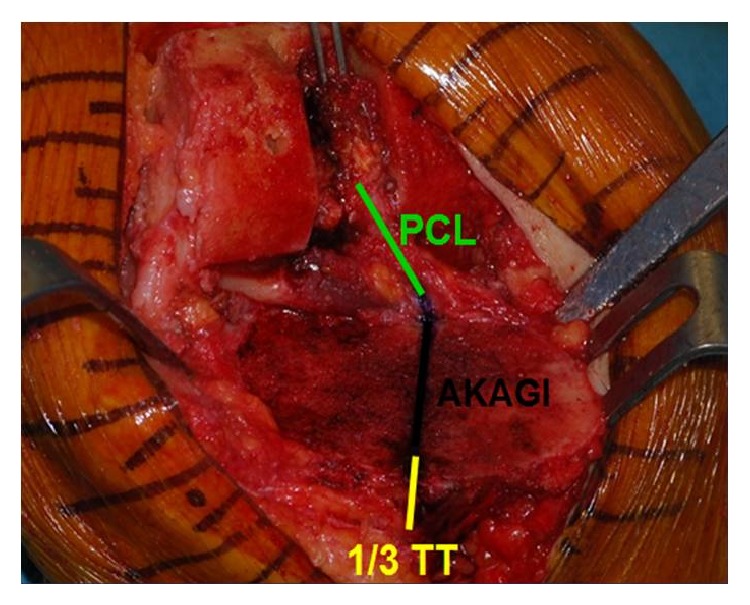
Anatomical landmarks for tibial component rotational alignment in TKA: posterior cruciate ligament (PCL), “Akagi line,” and medial third of the tibial tuberosity (1/3 TT).

Early TKA failures, related to tibial rotational malalignment, are characterized by anterior mechanism complications [6–8] and knee stiffness [9]. A standard tibial rotational reference is still controversial in the current literature. Few anatomical landmarks have been proposed in order to obtain an accurate rotational position of the tibial component, including the medial third of the tibial plateau [10], the “Akagi” line [11] (Figure 1), the central third of the tibial tubercle [12], and the posterolateral tibial corner [13]. In a previous study [14], the authors of the current study proposed a new method for positioning of the tibial component in TKA: we intended to ascertain if there was a more adequate way of orienting tibial components in TKA, starting from the fact that matching of orientation of two similar curves would be an easier definable landmark than a single anatomical point or a line (“Curve-on-Curve technique”). In that study, we demonstrated that the anterior tibial surface contour is a reliable landmark for correct tibial component rotational positioning, in TKA designs characterized by having a symmetric tibial baseplate, with respect to the “Akagi” line and the medial third of the tibial tubercle.

Several surgical techniques have been described to rotationally orient the tibial component in TKA, including the “self-range-of-motion” [1, 7] and the “maximizing tibial coverage” techniques [15]. The self-range-of-motion technique aligns the tibial component according to the rotational alignment of the femoral component during trial reduction with a “self-seeking method.” Because several morphological assessments concluded that contemporary tibial designs do not match global population morphology [16, 17], the industry's focus was shifted to achieve high coverage in many modern tibial designs, including asymmetric and even markedly anatomical designs. However, focusing solely on maximizing tibial coverage may lead to severe internal rotation errors [18].

The purpose of the current study was to test the reliability of the authors' previous tibial rotational alignment method (“Curve-on-Curve technique”) for accuracy using a strongly anatomical (right/left) tibial component design. Understanding the relationships between the two curves (anterior tibial baseplate and anterior tibial cortex) would guide surgeons when choosing an adequate tibial baseplate design thus reducing the risk of tibial malrotation. In order to validate the “Curve-on-Curve” surgical method on a different spectrum of tibial baseplate designs, the tibial rotational alignment of 2 modern tibial base designs (symmetric and anatomical) was assessed across two groups of patients.

The hypothesis of the current study is that, matching an anatomical landmark (anterior tibial cortex) with an industrial landmark (anterior contour of an anatomical tibial baseplate), the asymmetric tibial design leads to internal rotation when compared to the femoral TEA in a computed tomography (CT) study performed with the knee joint in full extension. The authors recognize that the validation of this hypothesis might lead to demonstrating that the surgical technique proposed (“Curve-on-Curve”) is not applicable to the broad spectrum of tibial baseplate designs.

## 2. Materials and Methods

The authors selected, after obtaining Institution Review Board (IRB) and patient consent, 80 consecutive patients affected by primary degenerative knee joint disease. All patients were scheduled for primary TKA. Preoperative diagnosis in this series was always osteoarthritis without previous history of trauma, previous surgery, or major knee dysplasia. Patients with documented mechanical varus or valgus malalignment were also included. Patients' mean age at surgery was 72 years (range, 60–81 years).

### 2.1. Total Knee Arthroplasty Component Designs

All patients received a posterior-stabilized (PS) fixed-bearing implant. Patient randomization was performed in the morning of the surgery and was accomplished with the use of a randomized numbers table. Patients with even numbers were assigned to one design and patients with an odd number were assigned to receive the other design. Patients were also blinded to the implant they received.

**Figure 2:**
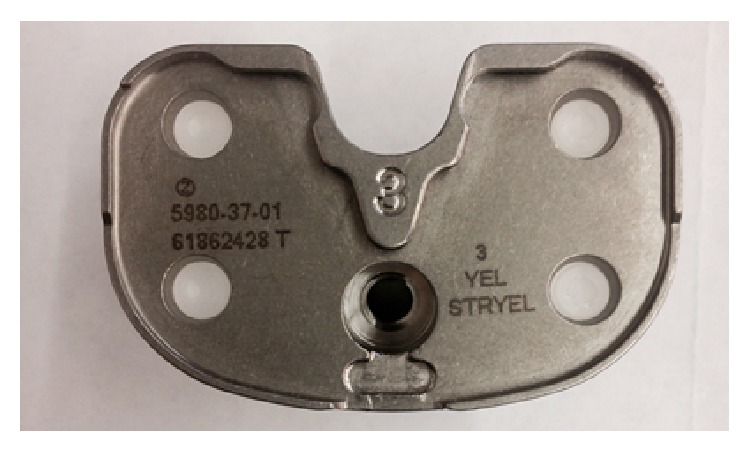
Tibial baseplate (Nex-Gen LPS, Zimmer, Warsaw, IN, USA): this component has a symmetric design.

**Figure 3:**
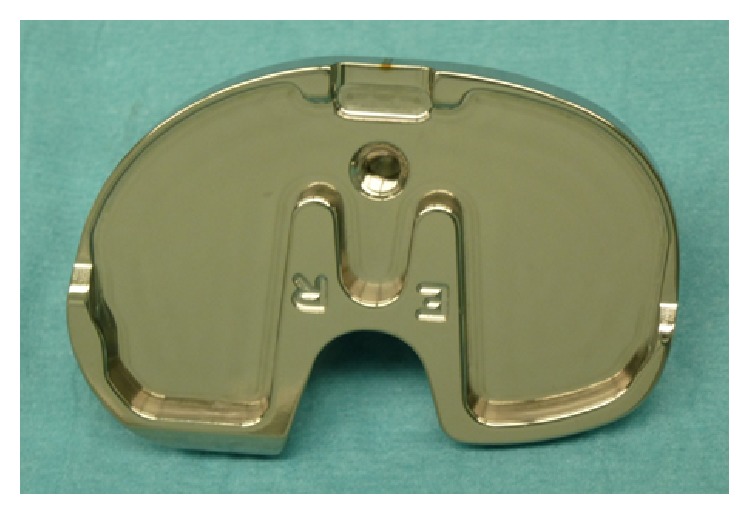
Right tibial baseplate (Persona, The Personalized Knee System, Zimmer, Warsaw, USA): this component is characterized by a strong anteromedial and posteromedial asymmetry.

Forty patients (Group A: Design 1) were randomly selected to receive a Nex-Gen legacy posterior substituting TKA implant (Nex-Gen LPS, Zimmer, Warsaw, IN, USA), characterized by a symmetric tibial baseplate (Figure 2). Forty patients (Group B: Design 2) received Persona, The Personalized Knee System TKA implant (Zimmer, Warsaw, IN, USA), characterized by having an anatomical tibial baseplate (Figure 3). All the available sizes in each component design were available for use at the time of surgery (Table 1).

### 2.2. Intraoperative Steps

**Figure 4:**
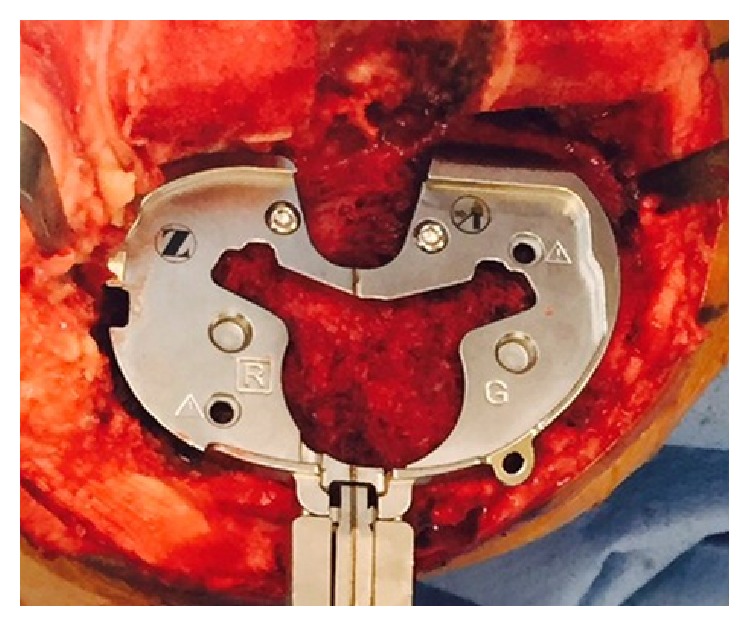
Right knee. Intraoperative view of the tibial baseplate positioning (Persona, The Personalized Knee System, Zimmer, Warsaw, USA). The rotational alignment of the tibial baseplate has been set according to the “Curve-on-Curve” technique [14]: the tibial component is set matching the tibial anterior cortex with its anterior contour.

The surgical approach in all cases included a standard midline skin incision and a medial peripatellar capsulotomy, avoiding lateral patellar retinacular releases. The chosen surgical technique was a combination of the “balanced gaps technique” [19] and the “measured resection technique” [20]: first, a rectangular extension gap was created; secondarily, the rotation of the femoral component was oriented according to the surgical transepicondylar (sTEA) axis. All implants were aligned on the coronal plane reproducing patient's neutral mechanical axis. All cemented PS femoral components (Designs 1 and 2) were aligned rotationally according to the patient's surgical TEA. The rotational alignment of all cemented tibial components (Designs 1 and 2) was set matching the contour of the tibial anterior cortex (Figure 4) (“Curve-on-Curve technique” for rotational alignment) [14]. All patellae were replaced using a “free hand technique” without cutting guides and tracking of the patella was checked using the “no thumb technique” [21]. A release of the deep lateral patellofemoral ligament without capsulotomy was performed if necessary. All patients followed identical postoperative rehabilitation protocol, including weight-bearing as tolerated beginning on postoperative day one.

Group 1 (Design 1) included 25 females (62.5%) and 15 males (38.5%). Average age was 72 years (range 60 to 81). Group 2 (Design 2) included 24 females (60%) and 16 males (40%). Average age was 71 years (range 66 to 80). Average preoperative anatomic alignment on standard anteroposterior knee view was 6.1° varus in Group 1 and 6.7° varus in Group 2 (range, varus 14°, valgus 11°).

### 2.3. Total Knee Arthroplasty Components Evaluation

**Figure 5:**
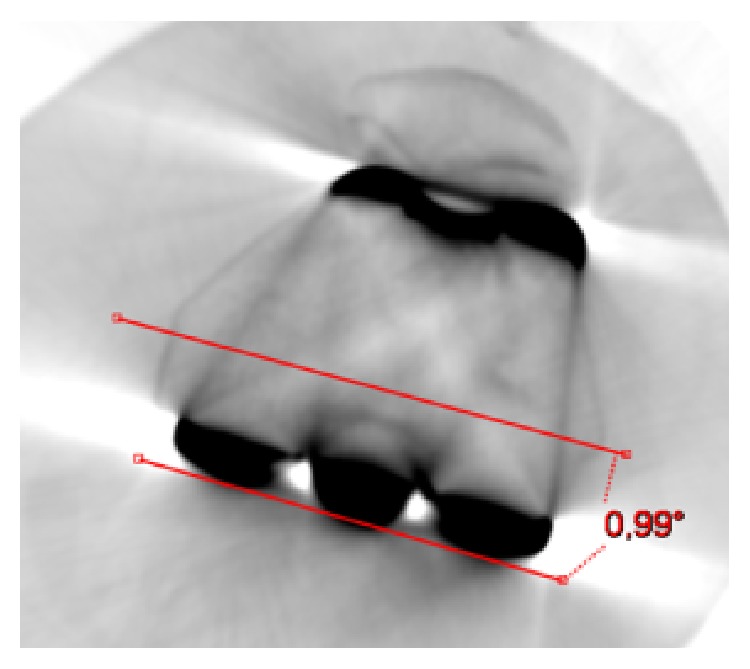
Left knee. Femoral component (Persona, The Personalized Knee System, Zimmer, Warsaw, USA) computed tomography (CT) axial view. The angle (PCA) between the surgical transepicondylar axis (sTEA) and the line of the posterior condyles is 0.99°.

**Figure 6:**
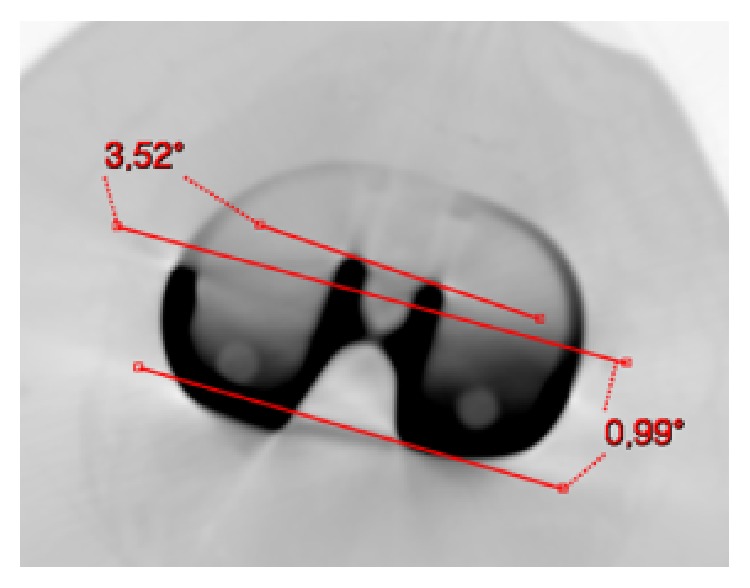
Left knee. Tibial component (Persona, The Personalized Knee System, Zimmer, Warsaw, USA) computed tomography (CT) axial view. Two angles were measured: (1) PCA (posterior condyles angle): angle between the projected surgical transepicondylar axis (sTEA) and the line of the posterior condyles (0.99°); (2) the angle between the projected sTEA and the mediolateral axis of the tibial component (3.52°). The mediolateral axis of the tibial baseplate has been identified through its “dwell points.” This tibial baseplate is externally rotated by 3.52° with respect to the projected femoral transepicondylar axis (sTEA).

All knees underwent computed tomography (CT) evaluation in the postoperative period utilizing a GE Healthcare system (Little Chalfont, UK). The scanning protocol included positioning the knee in full extension with the second metatarsal axis in a vertical position according to Berger et al. [22], which has been followed to obtain a reproducible knee position for all CT scans. All images were 2 mm in thickness and with 3 mm in reconstructive increments from the distal metaphysis to the tibial tubercle. A specific software (Sectra AB, Sectra, Sweden) was utilized for artifact suppression. On the best single femoral axial scan, the surgical femoral transepicondylar axis (sTEA) was selected and the femoral posterior condylar axis (PCA) was measured (Figure 5). At this point, the sTEA was transposed on the tibial axial cut where the mediolateral axis of the tibial baseplate was best identifiable through its “dwell points” (for the symmetric component) or the anterior axis of the polyethylene locking mechanism (for the asymmetric component); the rotation of the tibial component with respect to the sTEA was then measured (Figure 6).

The projected femoral sTEA has been proposed as a trustable anatomic landmark for rotational alignment of the tibial component by many authors, including the authors of the current study [14, 23–25]. Hutter et al. [26] showed that, independently of the chosen landmark for tibial rotational alignment, TKA increased laxity, decreased stiffness, and increased tibiofemoral motion during ROM but showed also little change based on the tibial alignment.

For the current study, customized software was created and used for analysis of the CT datasets. All selected axial images were evaluated independently by two observers (AG, GP), not involved in the original surgery. They independently repeated the entire measurement process, from point gathering to angles measurement for every knee part of the two study groups. The reproducibility of this method was then calculated by using Bland-Altman analysis for interobserver agreement. The rotational alignment measurements in the two groups were reported as an average value. Statistical analysis was performed using independent two-sample *t*-test. The calculated *p* value for statistical significance was set at 0.05.

### 2.4. Clinical Outcome Measurements

Clinical data from both study groups were assessed preoperatively and at 24-month minimum follow-up. The outcome assessments used were the Oxford Knee Score [27], the clinical and radiological Knee Society Score (KSS) [28], average ROM, and a satisfaction survey. A two-sample *t*-test comparing the two groups was performed.

## 3. Results

### 3.1. Symmetric Tibial Baseplate (Design 1)

All forty knees in this study group were available for radiological evaluation at follow-up. The rotational alignment measurement of the symmetric tibial baseplate with respect to the surgical TEA showed an average external rotation of 1.3° (minimum 5°; maximum −1°). All forty tibial components (100%) showed a rotation of 0 ± 5° with respect to the surgical TEA: 91% showed 0 ± 3° of rotation while 77.5% showed 0 ± 2° (Table 2; Figure 8). The tibial component appeared internally rotated at 1° with respect to the surgical TEA in 8 cases (20%). The average intraclass correlation coefficient was 0.927. The standard deviation value in this group was 1.826.

### 3.2. Anatomical/Asymmetric Tibial Baseplate (Design 2)

**Figure 7:**
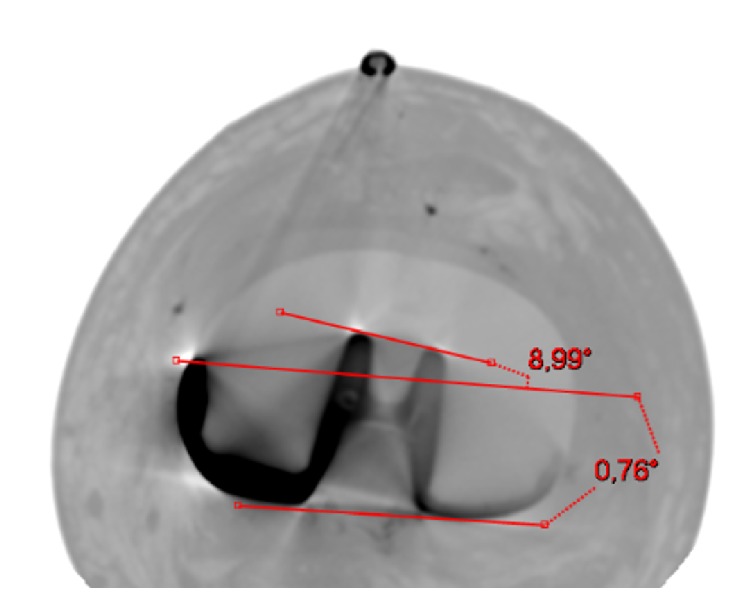
Left knee. Tibial component (Persona, The Personalized Knee System, Zimmer, Warsaw, USA) computed tomography (CT) axial view. Two angles were measured: (1) PCA (posterior condyles angle): angle between the projected surgical transepicondylar axis (sTEA) and the line of the posterior condyles (0.76°); (2) the angle between the projected sTEA and the mediolateral axis of the tibial component (8.99°). The mediolateral axis of the tibial baseplate has been identified through its “dwell points.” This tibial baseplate is externally rotated by 8.99° with respect to the projected femoral transepicondylar axis (sTEA).

**Figure 8:**
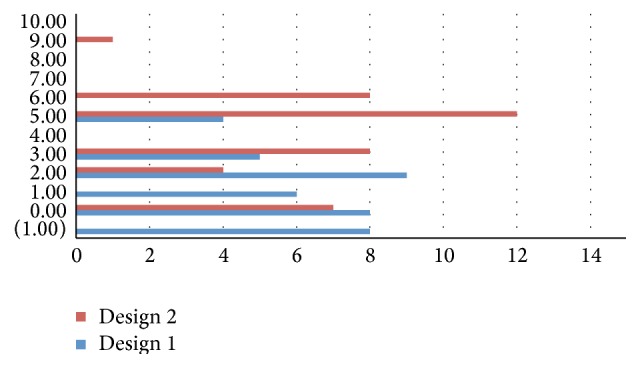
Tibial base rotation distribution for the two designs (Design 1: Nex-Gen Complete Knee Solution TKA implant; Design 2: Persona, The Personalized Knee System TKA implant; Zimmer, Warsaw, IN, USA). Vertical Axis (*y*): number of patients; horizontal axis (*x*): degrees of rotation (−1° to 10°) with respect to the reported surgical transepicondylar axis (TEA). The average rotation.

None of the patients in this study group were lost to follow-up. The rotational alignment measurement of the anatomical tibial baseplate with respect to the surgical TEA showed an average external rotation of 4.1° (minimum 0.4°; maximum 8.9°). Thirty-one tibial components (77.5%) showed a rotation between 0° and 5° with respect to the surgical TEA while 8 knees showed an external rotation of 6° and one knee had an external rotation of 8.9° (Figure 7). None of the Design 2 tibial baseplates demonstrated internal rotation with respect to the sTEA. The average intraclass correlation coefficient was 0.945. The standard deviation value in this group was 2.276 (Table 2; Figure 8). The calculated *p* value for statistical significance between Design 1 and Design 2 group was <0.0001.

### 3.3. Clinical Outcomes

All patients were available at 2-year follow-up: implant Group 1 (Design 1) patients showed a statistically significant increase in postoperative anterior knee pain (9% versus 3.4%; *p* = 0.008) and inferior average ROM (112° versus 122°; *p* = 0.0011) compared to implant Group 2 patients; differences in clinical and radiological KSS (*p* = 0.11), Oxford Score (*p* = 0.10), overall satisfaction rate, and survivorship in two years did not reach statistical significance. There were no revisions for any reasons in any study group.

## 4. Discussion

Rotational malalignment has been shown to be a major cause of premature failure and patient dissatisfaction after TKA [8, 29, 30]. While the transepicondylar axis is a well-recognized reference for the femoral rotational alignment [31–34] when the “measured resection” technique [20] is intraoperatively chosen for the rotational alignment of the femoral component, there is no consensus regarding a primary reference for the tibial rotational alignment. In fact, several surgical techniques, each one using a different anatomical landmark, have been proposed by previous studies as being accurate for tibial rotational alignment in TKA [23, 35, 36].

Few surgical techniques suggest the use of a single point as an intraoperative landmark for correct rotational alignment of the tibial component in TKA. Incavo et al. suggested aligning the anteroposterior axis of the tibial tray with a point close to the medial third of the patellar tendon [25]. Lützner et al., in a CT evaluation of 80 TKA, showed that referencing the tibial rotation on a line from the medial third of the tibial tubercle to the center of the tibial tray resulted in a better CT determined femorotibial rotational alignment than using the medial border of tibial tubercle as a landmark [10]. Matziolis et al. showed that the most prominent point of the tibial tubercle is more accurate than computer navigation for correct tibial component rotational alignment [37]. Ikeuchi et al. demonstrated, in a intraoperative and postoperative CT study, that using the medial border of the patellar attachment as tibial alignment landmark allows a more accurate tibial baseplate rotational alignment in respect to the range-of-movement technique [36]. Recently, Rossi et al., in a cadaveric study, validated the posterolateral tibial corner as a reliable reference landmark for tibial baseplate rotational alignment [13]: however, the identification of this landmark requires a complete exposure of the tibial plateau, which is often difficult to obtain in many knees.

Other surgical techniques suggested the use of an axis or a sagittal plane in place of a single-landmark for correct rotational alignment. Akagi et al. described a line perpendicular to the projected femoral TEA, starting at the medial third of the tibial tubercle and pointing at the middle of the posterior cruciate ligament tibial insertion [11] (Figure 1). Dalury proposed using a line from the midpoint between the tibial spines passing 1 mm medial to the medial border of the tibial tubercle [38]. Luo proposed the use of a line perpendicular to the posterior joint surface passing through the medial third of the tibial tubercle [39]. Unfortunately, many sagittal axes are not easily and reliably identifiable at surgery. Graw et al. showed high variability of several sagittal axes in relation to different tibial resection levels [24]. Nagamine et al. demonstrated that a sagittal anteroposterior axis was less reliable than the posterior condylar axis for use in tibial rotational alignment in TKA [40]. Siston et al. demonstrated that neither the axis technique nor the single-point reference technique establishes a correct tibial rotation alignment [41].

In a previous study [14], the authors of the current study hypothesized that the anterior tibial surface contour is a more reliable landmark for correct tibial component rotational positioning in TKA with respect to other axes or single-landmark references techniques: they showed that matching the contour of the tibial anterior cortex with a symmetric tibial baseplate yields a satisfactory rotational alignment between the femoral and tibial components, at least with the knee in full extension. Unfortunately, they were not able to demonstrate the same satisfactory rotational alignment during range-of-motion. Assessments of bone quality at the tibial resection level, performed by Bloebaum et al. [42], indicated weaker bone along the anterior cortex, predisposing tibial baseplates to anterior subsidence. Therefore, optimal coverage in this region may also be helpful to prevent loosening.

The most important finding of the current study was the discovery that tibial baseplate designs differ substantially in terms of rotational alignment when using identical anatomical landmarks. The authors recognize that this finding was only validated during CT evaluation of knees in full extension according to their arbitrary femoral sTEA. In Design 1 study group, all forty tibial components (100%) showed 0 ± 5° of rotation with respect to the surgical TEA: the average rotational alignment was 1.3° of external rotation. For Design 2, the practice of aligning the anterior contour of its anatomical tibial baseplate along the anterior tibial cortex led to an average external rotation of the tibial component by more than 4°: none of those tibial baseplates were internally rotated. More than 20% of those tibial baseplates showed an external rotation more than 5°.

Hypothetically, anatomic tibial component design offers increased morphological fit to the proximal tibia compared to nonanatomic designs by improving tibial coverage [15]. Several anatomical tibial baseplate designs have been proposed in order to increase the amount of tibial coverage with the goal of reducing the risk of aseptic loosening. Few surgeons, including Wevers et al. [43] and Hartel et al. [44], prefer the use of an asymmetrical/anatomical component maximizing tibial cover in order to provide stability and excellent load transfer in TKA and to mimic the asymmetry of the native tibia. Unfortunately, tibial coverage itself is not sufficient enough to guarantee a satisfactory tibial rotational alignment, leading to major malalignment errors when using modern tibial geometries [18, 45]. On the other hand, tibial coverage of classical designs rarely exceeds 78%. Several authors have proposed a minimum of 75% coverage for adequate fixation; however, this is based on mechanical data and the degree to which this is clinically relevant is still unknown [10]. Recently, Dai et al. [15], in a computer model study, suggested that anatomical designs correlate with better tibial coverage and contemporarily more accurate rotational alignment accuracy.

Regarding our differences in clinical outcome between the two study groups, the authors showed a decrease in the incidence of postoperative anterior knee pain and an increase in maximum knee flexion in Design 2 patients study group. Anyway, the current study has not been designed as a clinical study and the small number of patients involved and the short follow-up period must to be taken into consideration when evaluating the clinical outcomes of the current study. Design 2 is characterized by an extreme side-specific modularity (12 femoral sizes, 9 tibial sizes, 8 different tibial inserts, and 6 patellar sizes), a new J-curve femoral design, a deeper femoral groove, and a shorter anterior femoral flange when compared to Design 1. These differences might justify the differences in clinical outcomes at two-year follow-up.

Our study has several limitations. The main limitation is the use of a single anatomical landmark for tibial rotational alignment, not comparing the rotational alignment of our symmetric or asymmetric tibial components with different alignment intraoperative methods. It does not answer the question as to whether there is an overall optimal orientation of the tibial component during TKA. On the other side, we propone a reproducible method for tibial component rotational orientation when a symmetric tibial baseplate is utilized: our technique is based on the theory that an alignment between the projected sTEA and the mediolateral tibial baseplate axis is desirable [46, 47] when the knee reaches full extension. The authors acknowledge that many surgeons prefer a visual method based on an angular relationship between the tibia and the prosthetic baseplate and rotational incongruity during ROM may be unavoidable, but they strongly believe that rotational congruency between femoral and tibial components in full extension is extremely important. Based on the current study, it is unclear whether the tibial external rotation caused by aligning an asymmetric tibial baseplate according to the anterior tibial contour would lead to clinical complications because of lack of mid-to-long term functional results; the authors of the current study, Martin et al. [18], and Clary et al. [45] all concluded that setting rotational alignment by maximizing coverage should be avoided for all tibial base designs because of the risk of excessive internal rotation. This study also did not take into consideration the quality of bone that supported the tray and design-specific tibial resections were not investigated. Finally, the presence of osteophytes or significant bone defects may intraoperatively interfere with the resulting tibial resection, anterior profile, or placement of the tibial tray.

## 5. Conclusion

This study propones the anterior tibial contour as an adequate anatomical rotational alignment landmark for the use of symmetric tibial baseplates in total knee arthroplasty: the authors demonstrated a correlation between the position of a symmetric tibial component and the projected sTEA when the knee is locked in full extension. Our findings also suggest that the anterior tibial contour might not be the most accurate landmark for rotational alignment of the prosthetic components when an asymmetric tibial baseplate is intraoperatively chosen. Although many anatomical landmarks appear to be acceptable, they also might have the unresolvable problem in that rotational alignment between femoral and tibial components could not be completely synchronized during ROM because the alignment of each component is still determined separately in many current surgical TKA techniques.

## Figures and Tables

**Table 1:** Tibial component design families used in this study.

Design	A	B
Type	Symmetric	Anatomic
# sizes	10	9
ML size range (mm)	58.4–89.0	57.7–88.1
ML increments (mm)	0–8.0	3.0–5.1
AP increments (mm)	−1.5 to 4.0^a^	1.8–3.3^b^

^a^Negative increment (−1.5 mm) exists only between sizes 8 and 9.

^b^Increases asymmetrically between medial and lateral compartment.

**Table 2:** Computed tomography (CT) results. sTEA: surgical transepicondylar axis; ER: external rotation with respect to sTEA projected to the tibia; ^*∗*^statistically significant difference (*p* < 0.0001).

Tibial baseplate	*N*	Average ER^*∗*^	0 ± 5° sTEA^*∗*^	0 ± 3° sTEA^*∗*^	0 ± 2° sTEA^*∗*^
Symmetric (Design 1)	40	1.32° (5°/−1°)	100%	90%	77,5%
Asymmetric (Design 2)	40	4.15° (0,46°/8,99°)	77%	47,5%	27,5%
